# Racial disparities in cancer genetic counseling encounters: study protocol for investigating patient-genetic counselor communication in the naturalistic clinical setting using a convergent mixed methods design

**DOI:** 10.1186/s12885-023-11486-x

**Published:** 2023-10-16

**Authors:** Nao Hagiwara, Nadia Harika, Erin P. Carmany, Yongyun Shin, Susan Eggly, Shawn C. T. Jones, John Quillin

**Affiliations:** 1 Department of Public Health Sciences, University of Virginia, 200 Jeanette Lancaster Way, Charlottesville, VA 22903 USA; 2 Department of Pediatrics, Virginia Commonwealth University, 1008 East Clay Street, B-011 Box 980270, Richmond, VA 23219 USA; 3 Center for Molecular Medicine and Genetics, Wayne State University, 3127 Scott Hall, 540 E. Canfield Ave, Detroit, MI 48201 USA; 4 Department of Biostatistics, Virginia Commonwealth University, 830 East Main Street, One Capitol Square 718, Richmond, VA 23298 USA; 5 Department of Oncology, Wayne State University, Karmanos Cancer Institute, 4100 John R, Detroit, MI 48201 USA; 6 Department of Psychology, Virginia Commonwealth University, 806 West Franklin Street, 23284 Richmond, VA USA

**Keywords:** Cancer genetic counseling, Implicit bias, Explicit bias, Prejudice, Stereotyping, Racial healthcare disparities, Patient-provider communication, Clinical discussion, Black/African American

## Abstract

**Background:**

Despite decades of effort to reduce racial cancer disparities, Black people continue to die at higher rates from cancer than any other U.S. racial group. Because prevention is a key to the cost-effective and long-term control of cancer, the potential for cancer genetic counseling to play a central role in reducing racial cancer disparities is high. However, the benefits of genetic counseling are not equitable across race. Only 2% of genetic counselors self-identify as Black/African American, so most genetic counseling encounters with Black patients are racially discordant. Patients in racially discordant medical interactions tend to have poorer quality patient-provider communication and receive suboptimal clinical recommendations. One major factor that contributes to these healthcare disparities is racial bias. Drawing on findings from prior research, we hypothesize that genetic counselor providers’ implicit racial prejudice will be associated negatively with the quality of patient-provider communication, while providers’ explicit negative racial stereotypes will be associated negatively with the comprehensiveness of clinical discussions of cancer risk and genetic testing for Black (vs. White) patients.

**Methods:**

Using a convergent mixed methods research design, we will collect data from at least 15 genetic counseling providers, from two different institutions, and their 220 patients (approximately equal number of Black and White patients per provider) whose appointments are for a hereditary cancer condition. The data sources will include two provider surveys, two patient surveys, video- and/or audio-recordings of genetic counseling encounters, and medical chart reviews. The recorded cancer genetic counseling in-person and telehealth encounters will be analyzed both qualitatively and quantitatively to assess the quality of patient-provider communication and the comprehensiveness of clinical discussion. Those data will be linked to pre- and post-encounter survey data and data from medical chart reviews to test our hypotheses.

**Discussion:**

Findings from this multi-site study will highlight specific aspects of cancer genetic counseling encounters (patient-provider communication and clinical recommendations) that are directly associated with patient-centered outcomes (e.g., satisfaction, trust, genetic testing completion). Patient-provider communication and clinical recommendations are modifiable factors that can be integrated into current genetic counseling training curricula and thus can have immediate impact on genetic counseling training and practice.

## Background

Individuals with cancer are more likely to die from it if they are Black, [1] although hereditary cancer is distributed similarly across race [2, 3]. Because prevention is key to the cost-effective and long-term control of cancer, [4] the potential for cancer genetic counseling to play a central role in reducing racial cancer disparities is high. Genetic counselors address the medical, psychological, and familial concerns related to genetic disease, [5] including hereditary cancer. Yet, the benefits of genetic counseling are not equitable across race. Studies continue to show that Black patients with breast or colon cancer are less likely to discuss genetic evaluation or genetic counseling referral with their providers [6–8]. A systematic review also revealed racial disparities in genetic counseling experiences [9]. Despite a rapidly increasing discourse about, and recognition of, potential racial disparities in the genetic counseling process, only one study, to our knowledge, has directly compared Black and White patients [10]. In that study, Black women with breast cancer who had genetic counseling were less likely to have genetic testing. But, what happened during the genetic counseling session that may have led to this disparity was not examined.

Only 2% of genetic counselors in the US self-identify as Black/African American, while 90% self-identify as White American; [11] thus, genetic counseling encounters for Black patients will likely be racially discordant. This shockingly low number of Black genetic counselors (as a comparison, 5% of surgeons/physicians self-identify as Black/African American) [12] is a huge roadblock for the field of cancer genetic counseling to become an equalizer, because the quality of patient care tends to be poorer during racially discordant (vs. concordant) medical interactions in general [13–17]. One major factor contributing to such disparities is racial bias. Racial bias consists of prejudice (negative *feelings* toward racial minorities) and stereotyping (*beliefs* about racial minorities), both of which can operate at implicit and explicit levels [18, 19]. Bias at the implicit level reflects feelings and beliefs that are automatically and spontaneously activated; thus, it is relatively hard to control. In contrast, bias at the explicit level reflects a deliberate, effortful process and often takes into account social desirability concerns (e.g., desire to appear non-prejudiced). Both implicit and explicit bias reflect “true” feelings and beliefs; they simply reflect different psychological processes [19, 20]. In fact, consistent with prior research, [18–21] a recent study has shown that genetic counselors and genetic counseling trainees (we refer to them both as “genetic counseling providers” hereafter) displayed moderate pro-White/anti-Black implicit prejudice overall but no explicit prejudice [22]. Turning to implicit stereotyping, there was a trend for providers to associate White people with medical cooperativeness and Black people with medical non-cooperativeness. Finally, unlike physicians, genetic counseling providers, on average, reported explicitly that Black people are medically cooperative. However, they also reported that Black people are mistrustful of medical providers as well as of the healthcare system in general.

**Fig. 1:**
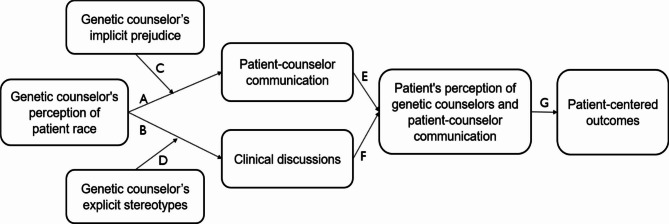
Our conceptual model of the role of genetic counselor’s bias in racial disparities in the genetic counseling processes

Distinguishing these forms of racial bias is important because they are associated with different aspects of healthcare. A recent review [23] provides evidence that providers’ greater implicit prejudice is associated with poorer quality patient-provider communication during racially discordant interactions, [24–28] which can further result in less patient satisfaction and trust in providers (Fig. 1) [29–31]. There is strong evidence that providers’ greater explicit negative stereotyping of Black patients is associated with suboptimal clinical recommendations for Black patients [21, 32, 33]. Drawing on findings from prior research, we developed a conceptual model highlighting genetic counselor bias, particularly implicit prejudice and explicit stereotyping, as major factors contributing to racial disparities in the genetic counseling process. We propose that patient-provider communication will be poorer for Black patients than for White patients (Path A) and that genetic counselors’ implicit prejudice toward Black people is negatively associated with the quality of patient-provider communication for Black patients, but not for White patients (Path C). We also propose that the content of clinical discussions will be lower in quality for Black patients than for White patients (Path B), and genetic counselors’ explicit negative stereotypes about Black people are negatively associated with the quality of clinical discussions for Black patients, but not for White patients (Path D). Finally, lower quality patient-provider communication and clinical discussions, in turn, will lead to Black patients’ negative perception of counselors and genetic counseling encounters (Paths E & F), ultimately contributing to racial disparities in patient-centered outcomes (Path G).

Research in healthcare disparities, social psychology, communication science, and our research all point to the role counselor bias may play in racial disparities in genetic counseling processes. Indeed, early studies of *simulated* genetic counseling encounters show that pro-White/anti-Black implicit prejudice leads to less individualized communication with patients [35] and may influence how genetic counselors help patients process emotional and cognitive information [36]. However, there are at least two major knowledge gaps that discourage us from starting with intervention research to address counselor bias. First, there has been no empirical documentation of the nature of such disparities in the *naturalistic* clinical setting. It is plausible that genetic counselors may communicate with patients differently in the naturalistic clinical setting than in a simulated one. More critically, the perspective and voice of actual patients are missing. Prior research shows standardized patient ratings of providers’ communication are different from actual patients’ ratings [37]. Notable, too, is that most research has not investigated trainees/students, yet trainees provide a significant proportion of genetic counseling. In the U.S., one-third of cancer genetic counselors report working at a university medical center, and one-third claim “student supervision” as one of their roles [11]. Thus, any generalizable research must include genetic counseling performed by supervised trainees.

Second, genetic counseling research has been hampered by both a paucity of process research and uncertainty about what outcomes are important and how they should be measured [38, 39]. Recently, the National Society of Genetic Counselors Research Task Force noted a critical need to answer basic questions such as “What are, and how do we measure, the psychological, behavioral, medical, interpersonal, and economic outcomes of genetic counseling?” [40] We argue that racial disparities in the genetic counseling process matter because they may impact clinically meaningful patient outcomes. We recognize that the definition of “clinically meaningful patient outcomes” varies case to case. Our conceptual model focuses particularly on patient-centered outcomes, including genetic testing completion, trust in and bond with providers, empowerment, and perceived self-efficacy. These outcomes may be predictive of patients’ subsequent health-related behaviors (e.g., lifestyle changes, healthcare utilization) [41, 42] and are applicable to a wide range of medical contexts.

## Methods

The overarching goal of this research is to compare and contrast the nature of cancer genetic counseling encounters and patient-centered outcomes between Black and White patients in the naturalistic clinical setting. We will test our hypotheses by addressing four specific aims: **Aim 1**) To compare/contrast the nature of patient-provider communication by patient race (Black vs. White); **Aim 2**) To compare/contrast the clinical discussion content by patient race; **Aim 3**) To quantify the role of each type of counselor bias in patient-provider communication (Aim 1) and clinical discussion content (Aim 2); and **Aim 4)** To quantify the role of patient-provider communication and clinical discussion content with patient-centered outcomes (trust, satisfaction, therapeutic alliance, empowerment, genetic testing completion). These aims will be achieved through an observational study with a convergent mixed methods research design. Table 1 provides an overview of the study design.

**Table 1:** An overview of the study designs

Aims	Data sources	Analyses	Goals
1	• Recorded encounters	Quantitative analyses of provider communication style and the overall quality of patient-provider communication Qualitative analyses of transcribed encounters for the four basic interaction structures	Identify specific aspects of patient-provider communication during cancer genetic counseling encounters that are different between Black and White patients
2	• Recorded encounters	Qualitative analyses of transcribed encounters for (1) the presence/absence of the key topics for risk communication and genetic test consent during cancer genetic counseling encounters that were identified by NCI; and (2) additional topics discussed during cancer genetic counseling encounters	Identify specific clinical discussion topics during cancer genetic counseling encounters that are different between Black and White patients
3	• Provider baseline survey • Patient baseline survey • Coded encounters	Quantitative analyses of associations between the four forms of provider bias and (1) patient-provider communication as well as (2) clinical discussion content that were identified in Aims 1 & 2	Identify specific aspects of patient-provider communication/clinical discussion topics that contribute to racial disparities in the cancer genetic counseling process and reflect provider bias
4	• Provider baseline survey • Patient baseline survey • Provider post-encounter survey • Patient post-encounter survey • Coded encounters	Quantitative analyses of associations between (1) patient-provider communication as well as (2) clinical discussion content that were identified in Aims 1 & 2	Identify specific aspects of patient-provider communication/clinical discussion topics that contribute to racial disparities in the cancer genetic counseling process and in patient-centered outcomes

Our conceptual model of racial disparities in the cancer genetic counseling process is applicable to many genetic counseling encounters. However, this research will focus on pre-test cancer genetic counseling encounters for both theoretical and methodological reasons. Traditional cancer genetic counseling involves at least two encounters with patients having genetic testing: [1] a pre-test session involving risk assessment and informed consent; and [2] a post-test session in which risks and medical management are re-assessed in light of genetic test results. More cancer genetic testing is being facilitated by non-genetics providers (e.g., direct-to-consumer testing, mainstream testing by oncology providers). In such cases, genetic counselors may shift to involvement only after testing is completed, and often only if patients test positive. However, we chose to focus on pre-test genetic counseling sessions for a number of reasons. First, the counseling elements in pre-test sessions are more uniform than in post-test sessions, especially in light of multigene panel testing consent, which, by necessity involves discussions that are often not specifically focused on individual genes and related medical management implications [43]. Focusing on pre-test cancer genetic counseling encounters also minimizes inherent variability across pre- vs. post-genetic testing contexts as well as across different areas/specialties of genetic counseling, providing greater ability to test our conceptual model. Second, for about nine out of 10 referred patients, genetic test results will be negative; [2] in most of these cases, the risk assessment and medical management recommendations content will remain largely unchanged. Finally, studies show that genetic testing completion is lower when pre-test counseling is provided by genetics providers, [44] compared with non-genetics providers. By focusing on pre-test genetic counseling, we can gain insight into predictors of genetic testing completion, especially whether racial bias plays a role. After validating our conceptual model in the context of pre-test cancer genetic counseling, our future work can examine whether this model is generalizable to other genetic counseling contexts. Such empirical evaluation of the generalizability of the conceptual model will further contribute to theory development.

### Participants

We will recruit *at least* 15 genetic counseling providers (both genetic counselors and MS genetic counseling trainees) and 220 patients (approximately equal number of Black and White patients for each provider) from two different institutions that serve diverse patient populations. The eligibility criteria for *genetic counseling providers* are that they are either American Board of Genetic Counseling certified genetic counselors or trainees in at least the second rotation for cancer genetic counseling. Even if there are too few male or minority providers to conduct meaningful inferential statistics, we will not exclude anyone based on sex/gender or race/ethnicity. Inclusion of all providers may be hypothesis generating for future work. The eligibility criteria for *patients* are: 1) self-identify as either Black/African American or White American; 2) ≥ 18 years old; 3) have an upcoming cancer genetic counseling appointment primarily focused on assessment of, and/or counseling for, a hereditary cancer condition with one of the participating providers at any of the recruitment sites; and 4) be able to comprehend all documents in English, written at a 6th grade reading level.

### Procedures

#### Data collection

Genetic counseling providers who meet the eligibility criteria and provide written consent will complete a 15-min baseline survey online before their first recorded appointment with a participating patient. The provider baseline survey is designed to assess all four types of bias as well as covariates that are likely to be associated with patient-provider communication, clinical discussion, and/or patient-centered outcomes, including demographic information, professional information, and prior training experiences. Genetic counseling providers will also complete a 1-min post-encounter survey after each genetic counseling encounter with a participating patient. Patients who meet the eligibility criteria will complete a 45-min baseline survey over the phone following the consent and HIPAA authorization and before the scheduled appointment with their participating genetic counseling provider. The patient baseline survey is designed to assess covariates that are likely to predict patient-provider communication, clinical discussion, and/or patient-centered outcomes. After their appointment, patients will complete a 5-min post-encounter survey. Table 2 provides an overview of the variables assessed in the surveys.

**Table 2:** An overview of the variables assessed in the surveys

	Genetic counseling providers	Patients
**Baseline**	• Demographic information • Professional information • Implicit racial prejudice • Explicit racial prejudice • Implicit racial stereotyping • Explicit racial stereotyping	• Demographic information • Socioeconomic information • Perceived racial discrimination • General trust in genetic providers • General satisfaction with healthcare
**Post-encounter**	• Therapeutic alliance	• Therapeutic alliance • Provider-specific trust • Provider-specific satisfaction • Perceived empowerment
**Medical chart**		• Genetic test uptake

On the day of the appointment, the patient-genetic counseling provider clinical encounter will be video- and audio-recorded. *For patients who have appointments in-person*, we will use two GoPro10 cameras (one focusing on the patient, another focusing on the provider) mounted on the wall with Velcro. These portable devices are designed to minimize intrusiveness while capturing patient-provider communication. We will attach the two cameras to the walls of the consultation room prior to the patient entering. The genetic counseling provider and patient will meet as scheduled while we control the recording from outside of the room using a GoPro device called The Remote [45]. Additionally, a digital voice recorder will capture audio of the interaction to be used in the unlikely event that the video fails. After each encounter, the genetic counseling provider will complete a 1-minute post-encounter survey, and the patient will be escorted to another room to complete a 5-minute post-encounter survey. Both the provider and the patient will be given three options to complete the survey: [1] paper and pencil, [2] a tablet with optional stylus, and [3] a QR code to scan and complete the survey on a personal device.

*For patients who have appointments via telehealth*, we will record the encounters locally on a laptop using Camtasia® screen capture software [46]. We will join the virtual appointment with both camera and microphone turned off. The patient and provider will meet as they typically would, and we will capture a screen recording of the appointment using Camtasia. Immediately after the telehealth encounter, the provider will receive a link to the provider post-encounter survey, and the patient will receive a phone call to complete the patient post-encounter survey over the phone. Providers will receive $30 after completing the provider baseline survey, and patients will receive $30 after completing the post-encounter survey.

Finally, we will review electronic medical records to assess whether patients agreed to take a genetic test after the recorded pre-test genetic counseling session. We will also assess genetic testing completion up to 6 months after the recorded genetic counseling session by reviewing patient electronic medical records (0 = test not completed, 1 = test completed).

#### Analysis of recorded genetic counseling encounters

We are going to use recorded genetic counseling encounters to assess both the observed quality of patient-provider communication and topic comprehensiveness of clinical discussion. This will be done by both observing recordings directly and analyzing verbatim transcripts of the recordings.

#### Patient-provider communication quality

The quality of patient-provider communication will be analyzed in two ways: global affect and linguistic patterns. To assess global affect, we will observe recordings and rate genetic counseling providers’ communication style by using the Global Affect Ratings from the Rotor Interaction Analysis System (RIAS) [47]. RIAS is the most commonly used method for coding genetic counseling encounters, [35, 36, 48] so use of RIAS enables us to directly compare our findings to those from prior research. We will use the Global Affect Ratings because the ratings reflect not only verbal behaviors (i.e., the context of speech) but also nonverbal (e.g., eye gaze, body posture, hand gestures) and paraverbal (e.g., tone, pitch, speed) behaviors, which tend to reflect an individual’s implicit prejudice [18–20]. The Global Affect Ratings consists of 14 categories: responsiveness/engagement; dominance/assertiveness; anger/irritability; anxiety/nervousness; hurried/rushed; friendliness/warmth; interest/attentiveness; depression/sadness; sympathy/empathy; emotional distress/upset; respectfulness; interactivity; scriptedness; and spontaneity. We will add a rating to assess the overall quality of patient-provider communication (e.g., smooth, awkward, good quality).

Each encounter will be rated by 15 racially diverse female coders. Coders will be all female because prior research has shown that women are more likely than men to make more accurate judgments of others’ characteristics [49]. Research has shown that over-thinking and over-reasoning can often disrupt the accuracy of global judgements and that people usually make better judgments when they do not try to explain their judgments [49–51]. Thus, we will not train coders to identify specific behaviors that might play role in making global judgments (e.g., smiling, frowning, eye contact). We will simply instruct them to report their impressions by using the full scale [24]. Coders will view encounters one by one and provide their ratings first for the 14 categories and then the additional items to assess overall patient-provider communication. The presentation order of the encounters will be randomized across coders. We will compute a composite score for each affective category and the overall patient-provider communication by averaging the ratings across coders. This composite score will be used in the statistical analysis.

To assess *the linguistic patterns of* patient-provider communication, we will both observe recordings and analyze transcripts. Specifically, we will examine genetic counseling providers’ linguistic patterns that reflect constructs associated with levels of implicit prejudice during racially discordant interactions in prior research, including social dominance, [52, 53] intergroup anxiety, [54–56] and avoidance (or lack of engagement) [57–59]. For social dominance, we will code: [1] patient-provider talk time ratio [25, 60]; [2] frequency of first person plural pronouns [26]; [3] turn-taking ratio [61, 62]; and [4] the number of interruptions and overlapping speech [63, 64]. For intergroup anxiety, we will code: [1] frequency of anxiety-related words (e.g., worried, afraid, nervous); [26] and [2] frequency of hesitation markers (e.g., uh, uhm, umm). For avoidance, we will code: [1] encounter length; and [2] frequency of back-channeling responses (e.g., Okay, yes, ah huh) [65].

Frequencies (i.e., first person plural pronouns, anxiety-related words, hesitation markers, back-channeling responses) will be assessed with the Linguistic Inquiry and Word Count (LIWC) software [66]. Each recording will be first transcribed verbatim, and then the verbatim transcripts will be further edited using the guidelines specifically developed for text analysis using LIWC [67]. Finally, the edited transcripts will be divided into two documents: patient vs. provider speech. The LIWC computes the percentage of use of words in more than 70 + word categories; it also enables researchers to create new word categories. For the current analyses, we will use both existing (i.e., pronouns, affective processes) and new (i.e., hesitation markers, back-channeling responses) categories.

The remaining coding will be conducted manually in steps. First, we will develop a linguistic codebook, which provides coding procedures, coding rules, descriptions, and examples of each linguistic pattern. Next, two senior research personnel will code a few transcripts and refine the linguistic codebook, and train two coders to apply the codes. The coders will then code all transcripts independently, while we check interrater reliability every 10 transcripts to prevent coder drift [68]. We will use the thresholds of *ICC* = 0.80 on 95% of codes [69]. We will use NVivo software to catalog and sort the coded data. Finally, we will take the findings from the analysis of linguistic patterns and create quantifiable units (Table 3) to be used in the statistical analysis.

**Table 3:** An overview of the variables, measurements units, and regression types

Constructs and corresponding linguistic patterns		Units of measurement	Regression type
**(1) Social dominance**			
	Patient-provider talk time	Duration	Linear
	First person plural pronouns	Proportion	Linear
	Turn-taking	Frequency	Poisson
	Interruptions and overlapping speech	Frequency	Poisson
**(2) Interaction anxiety**			
	Anxiety-related words	Proportion	Linear
	Hesitation markers	Proportion	Linear
**(3) Avoidance (Lack of engagement)**			
	Encounter length	Duration	Linear
	Back-channeling responses	Proportion	Linear

#### Topic comprehensiveness of clinical discussion

Next, we will assess the extent to which topics discussed during clinical encounters are consistent with key topics included in the National Cancer Institute’s (NCI) Cancer Genetics Risk Assessment and Counseling (PDQ®)-Health Professional Version [70]. To do this, two genetic counselors on our team will create a topic codebook based on the PDQ®-Health Professional Version. The codebook will provide coding procedures and rules as well as descriptions and examples of each topic. While the codebook is being developed, two coders will unitize the verbatim transcripts into discrete topics or focal ideas/themes. The coders will be first trained on the unitization with a few transcripts before they start unitizing all transcripts. They will meet every 5 transcripts to discuss and solve discrepancies in the identified thought units. After all the verbatim transcripts are unitized into discrete topics, two coders will be trained on the codebook until they achieve consensus and then code all transcripts independently, while meeting every 10 transcripts to discuss discrepancies and reach consensus. We will use consensus estimates for two reasons. Methodologically, discussion topics are discrete categories [71]. Clinically, providers are expected to discuss with patients each topic clearly and with no ambiguity. Finally, for each encounter, we will create dichotomous (a given topic discussed/not discussed) and continuous (the total amount of time spent on discussing a given topic in seconds) outcomes.

We will also explore additional topics beyond those specifically included in the PDQ®-Health Professional Version. To identify these additional topics, the genetic counselors on our team will independently review all units with no assigned codes. Next, they will discuss discrepancies in identified topics and collectively refine and organize them into higher-level categories. We will create an additional topic codebook that only includes topics that were identified by the two genetic counselors but not currently in the PDQ®-Health Professional Version. After being trained on the codebook, two independent coders will code all transcripts independently. Any discrepancy in coding between the two coders will resolved by discussion and reaching consensus. Finally, for each encounter, we will create dichotomous (a given topic discussed/not discussed) and continuous (the total amount of time spent on discussing a given topic in seconds) outcomes.

## Measures

### Genetic counseling provider baseline survey

#### Demographic information

We will assess self-reported age, race, ethnicity, gender, and sexual orientation.

#### Professional information

We will assess status (genetic counselor vs. genetic counseling trainee), years in practice (genetic counselor only), years at the current institution (genetic counselor only), or year in training (genetic counseling trainees only).

#### Prior experience with cultural competency training

Genetic counseling providers will be asked to indicate whether and when they most recently *participated in* as well as *led* training related to cultural competency. Providers who have participated in and/or led such training will be further asked to respond to the following set of four items: [1] the main goal(s) of the most recent training they participated in/led (free text); [2] whether the training was part of their required duties or curriculum (yes/no); [3] whether the most recent training was the first training they have had/led in cultural competency and related topics (yes/no); and [4] approximately how many hours of cultural competency-related training they have completed/led over the last 2 years (in hours). Finally, all providers will be asked to rate their ability to provide culturally competent genetic counseling (1: Poor to 5: Outstanding).

#### Prior experience with communication skills training

Similarly to the assessment of prior experience with cultural competency training, genetic counseling providers will first be asked to indicate whether and when they participated in and/or led communication skills training most recently. Those who have participated in and/or led communication skills training will be further asked to respond to the following set of four items: [1] the main goal(s) of the most recent training they participated in/led (free text); [2] whether the training was part of their required duties or curriculum (yes/no); [3] whether the most recent training was the first communication skills training they have had/led (yes/no); and [4] approximately how many hours of communication skills training they have completed/led over the last 2 years (in hours). All providers will be also asked to rate their communication skills (1: Poor to 5: Outstanding).

#### Prior experiences with Black patients

We will assess genetic counseling providers’ prior experiences with Black patients with two items: [1] how often they meet with Black patients in their practice or as part of their training (1: Never to 6: All the time); and [2] how they would rate their communication skills with Black patients (1: Poor to 5: Outstanding).

#### Implicit racial prejudice

Implicit racial prejudice will be assessed with the well-validated Race Implicit Association Test (Race IAT) [72, 73]. Individuals classify items into four categories: two racial groups (White vs. Black) and two evaluations (good vs. bad), which are presented in pairs. Participants respond more quickly when the racial group and evaluations mapped onto the same response are strongly associated than when they are weakly associated. In the Race IAT, two racial groups are represented by 12 photographs of faces, and two evaluative categories are represented by 16 words (8 good words: marvelous, superb, pleasure, beautiful, joyful, glorious, lovely, wonderful; and 8 bad words: tragic, horrible, agony, painful, terrible, awful, humiliate, and nasty). The Race IAT is scored by computing a *D* score that ranges from − 2.0 to 2.0 (the interitem consistency mean *α’*s = 0.78) [74].

#### Explicit racial prejudice

We will use a widely-used, well-validated “feeling thermometer,” [75] in which participants rate their feelings toward certain social groups using a scale ranging from 0 (very cold) to 100 (very warm) [76]. We will create composite explicit pro-White/anti-Black prejudice scores, which mirror *D* scores in the IAT, by subtracting a score for Black Americans from a score for White Americans. Greater positive values indicate explicit preference for White Americans over Black Americans while greater negative values indicate explicit preference for Black Americans over White Americans. A value of zero indicates no explicit preference between Black and White Americans.

#### Implicit racial stereotyping

Implicit racial stereotyping will be assessed with the medical cooperativeness IAT, the commonly used implicit measure of racial stereotyping in racial health disparities research [77, 78]. The task is the same as the Race IAT except that two evaluative categories (good vs. bad) are replaced by two stereotyping categories (medically cooperative vs. medically uncooperative). More specifically, 16 words that are to be categorized into evaluations (good vs. bad) are replaced by 12 words that are to be categorized into stereotypes (6 words associated with cooperativeness: willing, cooperative, compliant, reliable, adherent, helpful; and 6 words associated with uncooperativeness: reluctant, doubting, hesitant, apathetic, resistant, and lax). *D* scores will be computed using the same algorithm as for the Race IAT. Greater positive values indicate implicit association of White (vs. Black) Americans with medical cooperativeness, while greater negative values indicate implicit association of Black (vs. White) Americans with medical cooperativeness.

#### Explicit racial stereotyping

Genetic counseling providers will be asked to complete a 32-item measure that is designed to assess racial stereotypes in general (16 items for Black Americans, and 16 items for White Americans; e.g., unintelligent, lazy, low socioeconomic status) and a 34-item measure that is designed to assess stereotypes specifically in the medical context (17 items for Black Americans, and 17 items for White Americans; e.g., medical cooperativeness, health literacy, motivation), [21] which were adapted from previous studies of racial stereotyping [31–34]. In both measures, genetic counseling providers will be instructed to read statements and indicate the extent to which each statement describes characteristics of Black vs. White Americans, on a Likert scale that ranges from 1 (very untrue) to 6 (very true). Following the procedure used in prior research, a composite score will be computed for each general- and medicine-specific stereotype. Specifically, we will first reverse-code negatively worded items and compute the average score for each racial category. Next, we will subtract the average score for Black Americans from the average score for White Americans. Greater positive values indicate explicit association of White Americans (vs. Black Americans) to more positive stereotypes, while greater negative values indicate explicit association of Black Americans (vs. White Americans) to more positive stereotypes.

### Patient baseline survey

#### Demographic information

We will assess self-reported age, race, ethnicity, gender, pronouns they use, sexual orientation, U.S. citizenship, height and weight (to compute BMI), lifestyle cancer risk factors (e.g., smoking, alcohol use, physical activity), marital status, education, income, the number of people in household, personal and family cancer history, health insurance, and lifestyle behaviors (e.g., diet, physical activity, smoking, and alcohol consumption).

#### Perceived racial discrimination

Patients will be asked to complete two well-validated measures of perceived racial discrimination that have been used in health research and social psychology research previously. The first is the Brief Perceived Ethnic Discrimination Questionnaire-Community Version (*α* = 0.87) that assesses both daily and lifetime experience of multiple forms of discrimination (e.g., exclusion, stigmatization, threat) in multiple domains (e.g., work, public places) [79]. Participants will be asked to use a scale ranging from 1 (Never) to 5 (Very often). The second measure assesses the perceptions of racial discrimination at both personal and group level [80, 81]. More specifically, five items are designed to assess the degree to which patients have experienced discrimination personally (*α* = 0.84), and three items are designed to assess the degree to which patients perceive other members of their racial group in general experience racial discrimination (*α* = 0.84). Both subscales will be assessed by using response items that range from 1 (Strongly disagree) to 5 (Strong agree).

#### General trust in genetic counselors

We will assess patients’ expectation about their trust in general counselors within the specific health system with a modified version of the Wake Forest Physician Trust Scale, which has been found to have better internal consistency (*α* = 0.93, test-retest reliability = 0.75), validity, discriminability, and scale distribution as compared to other trust scales [82, 83]. To account for patients having never met with a genetic counselor, they will be instructed to indicate how they expect to feel regarding trust in their counselor. The response items will range from 1 (Strongly disagree) to 5 (Strongly agree).

#### General satisfaction with healthcare

We will use a modified version of the Patient Satisfaction Questionnaire Form III [84] to assess patients’ satisfaction with the specific *healthcare system* in general, as opposed to satisfaction with genetic counselors within the specific healthcare system, because the vast majority of patients will have had no prior experience with genetic counselors. In Particular, we will use three subscales: general satisfaction (*α* = 0.88), interpersonal aspects (*α* = 0.82), and communication (*α* = 0.82). The response items will range from 1 (Strongly disagree) to 5 (Strongly agree).

#### Perceived empowerment

We will assess perceived empowerment with the Genomics Outcomes Scale (GOS) [85]. The GOS is a six-item scale measuring patient empowerment following genetic counseling. This scale was derived from and is highly correlated (*r* = .84) with the longer Genetic Counseling Outcomes Scale [86]. The response items range from 1 (Strongly disagree) to 5 (Strongly agree).

#### Perceptions of genetic testing

Patients will complete the 14-item Attitudes Toward Genetic Testing (ATGT) scale (*α* = 0.73) [87]. It assesses perceptions of anxiety/conflict, discrimination, risk information, and prevention/surveillance relevance of cancer genetic testing and is suitable for providers, patients, and the general public. Again, the response items range from 1 (Strongly disagree) to 5 (Strongly agree).

#### Decisional conflict

Patients will complete a 16-item Decisional Conflict Scale (DCS) (*α* = 0.78 to 0.92), [88] modified for the context of hereditary cancer testing. The scale captures patients’ uncertainty about their genetic test decision, what factors contribute to that decision, and how effective they perceive they are in making a decision. Again, the response items range from 1 (Strongly disagree) to 5 (Strongly agree).

### Genetic counseling provider post-encounter survey

#### Therapeutic alliance

We will assess a provider’s perception of the strength of the relationship with a patient using the Working Alliance Inventory-Short Form Revised (WAI-SR) (Therapist). This 12-item measure assesses three domains: goals of the genetic counseling session, tasks to achieve those goals, and bond between the provider and patient. The WAI-SR has high internal consistency (*α* = 0.91) and correlates well with other measures of therapeutic alliance [89]. The response items will range from 1 (Strongly disagree) to 5 (Strongly agree).

#### Decision about genetic testing

Genetic counseling providers will be asked to indicate whether their patient made a decision about genetic testing during the genetic counseling session using the following options: [1] yes, and they decided to take genetic testing; [2] yes, and they decided not to take genetic testing; [3] no, they need more time to think about it; and [4] no, I did not offer genetic testing.

### Patient post-encounter survey

#### Decision about genetic testing

Patients will be asked to indicate whether they made a decision about genetic testing during the genetic counseling session using the following options: [1] yes, and I decided to take genetic testing; [2] yes, and I decided not to take genetic testing; [3] no, I need more time to think about it; and [4] no, I was not offered genetic testing.

#### Therapeutic alliance

Similarly to genetic counseling providers, the patient’s perception of the strength of the relationship with a genetic counseling provider will be assessed with WAI-SR (Client) [89].

#### Provider-specific trust

We will assess patient trust in the specific genetic counseling provider they have just met with a modified version of the Wake Forest Physician Trust Scale [83]. Items will be modified to reflect the specific provider a patient saw during their encounter.

#### Provider- and encounter-specific satisfaction

Patient satisfaction with the specific genetic counseling provider they have just met as well as with the counseling session they have just completed will be assessed with the 6-item Genetic Counseling Satisfaction Scale (GCSS) using a 5-point scale ranging from 1 (Strongly disagree) to 5 (Strongly agree). The GCSS assesses the extent to which patients feel the encounter was valuable and whether the provider understood the patient’s concerns. It has demonstrated excellent reliability (*α* = 0.90) among breast cancer genetic counseling patients [90] and has been used recently to assess satisfaction among Black patients [91] as well as patients receiving telegenetic counseling [92].

#### Perceived empowerment

We will use the same GOS [85] as in the patient baseline survey to assess perceived empowerment.

#### Perceptions of genetic testing

Perceptions of genetic testing will be assessed with the same ATGT scale [86] as in the patient baseline survey.

#### Decisional conflict

Patients’ decisional conflict related to hereditary cancer testing will be assessed with the same DCS [88] as in the patient baseline survey.

### Statistical analysis

A Monte Carlo simulation with 5,000 simulated datasets revealed that we can achieve adequate power (0.80) to detect a small-to-moderate effect (*Cohen’s d* = 0.2) of provider implicit racial prejudice on provider communication styles (with 10 provider factors and 5 patient factors in a model) with a total of 220 patients (the number of providers is not specified because the model is not multilevel). The sample size of 15 providers was determined by a follow-up Monte Carlo simulation that does not take into account patients nested within providers. This number is also consistent with our prior work that was able to detect the role of physicians’ implicit racial prejudice in patient-physician communication with 14–18 physicians [24–27].

Aim 1 (to compare/contrast the nature of patient-provider communication by patient race) examines Path A in our conceptual model. To test Path A, first, we will examine a numerical and graphical summary of descriptive statistics that will inform the distribution of the 15 outcomes (14 categorical variables and the overall patient-provider communication) and that may require data transformation. Then, we will conduct two separate analyses for communication and linguistic patterns. To examine how genetic counseling providers’ communication style differs by patient race, we will identify covariates by conducting bivariate correlations among all 15 outcomes and factors that were assessed in the baseline surveys (e.g., provider/patient demographics including sex, provider professional characteristics, patient perceived discrimination). Next, we will estimate a linear regression model for each outcome with the main effect of patient race, the main effect of care modality, a two-way interaction between patient race and care modality, and covariates identified as highly correlated with the outcomes. We will use GEE (which controls for the dependence in outcomes due to patients nested within providers) in these regressions, instead of multilevel modeling (which models the nonindependence in data), because there will not be enough upper-level units (i.e., counselors) to model random effects in our data. We will use the Bonferroni correction [93, 94] to address family-wise errors due to multiple comparisons. To examine which specific linguistic patterns differ by patient, we will conduct analyses in similar steps described above. First, we will identify highly correlated covariates by computing bivariate correlations among all quantified linguistic patterns and factors assessed in the baseline surveys. Next, we will conduct a regression for each quantified linguistic pattern with the main effect of patient race, the main effect of care modality, a two-way interaction between the two, and covariates using GEE and the Bonferroni correction. Type of regressions will depend on the measurement units.

Aim 2 (to compare/contrast the clinical discussion content by patient race) examines Path B in our conceptual model. We will examine which topics significantly differ between Black and White patients. First, we will identify covariates that may impact clinical discussion content by computing bivariate correlations among all topics and factors assessed in the baseline surveys. All significant factors will be included in the main analyses as covariates. In the main analyses, we will conduct a logistic regression (the dichotomous outcome) and a linear regression (the continuous outcome) for each topic with the main effect of patient race, the main effect of care modality, a two-way interaction between the two and covariates using GEE and the Bonferroni correction.

Aim 3 (to quantify the role of each type of counselor bias in patient-provider communication and clinical discussion content) examines Paths C and D in our conceptual model. Analyses will be carried out in three steps. In Step 1, we identify covariates associated with patient-provider communication or clinical discussion content. We will compute correlations among all facets of patient-provider communication/clinical discussion topics identified in Aims 1 & 2 and factors assessed in the baseline surveys (e.g., provider/patient demographics, provider professional characteristics, patient perceived discrimination). In Step 2, we compute partial correlations between all forms of provider bias and aspects of patient-provider communication/clinical discussion topics identified in Aims 1 & 2, controlling for covariates that were significantly associated with any of the provider behaviors. In Step 3, we conduct two regressions for each of the specific aspects of patient-provider communication and clinical discussion topics that remain statistically significant in Step 2. One regression will focus on the role of provider prejudice and will consist of four main effects (patient race, provider implicit prejudice, provider explicit prejudice, care modality), all six combinations of two-way interactions, all four combinations of three-way interactions, and one four-way interaction among all predictors. The second regression model will focus on the role of provider stereotyping and will consist of four main effects (patient race, provider implicit stereotyping, provider explicit stereotyping, care modality), all six combinations of two-way interactions, four combinations of three-way interactions, and one four-way interaction among all predictors. The type of each outcome, summarized in Table 4, will determine the regression model to analyze. The regressions will also include the same set of covariates as in Step 2. All four types of provider bias will be grand-mean-centered. We will use GEE and the Bonferroni corrections in analyses. The provider communication styles that remain statistically significant in Step 3 are considered as key communication styles that contribute to racial disparities in the cancer genetic counseling process and reflect provider bias.

**Table 4:** An overview of the outcomes and analyses for each aspect of patient-provider communication and clinical discussion content

Outcome types	Variables	Analytical approaches
Continuous: Likert scale	*Patient-provider communication* (Provider communication style; The overall quality of patient-provider communication)	Linear regression with GEE
Continuous: Frequency	*Patient-provider communication* (Each type of silence; Each type of adjacency pairs; Each type of problems)	Poisson regression with GEE
Continuous: Proportion	*Patient-provider communication* (Topics initiated by providers; Primary units allocated to providers; Silence followed by provider talks; Repairs initiated by providers; Problems repaired by providers)	Linear regression with GEE
Continuous: Duration	*Patient-provider commu*nication (Each type of silence) *Clinical discussion con*tent (The amount of time spent on discussing a given topic)	Linear regression with GEE
Dichotomy	*Clinical discussion content* (The presence/absence of a given discussion topic)	Logistic regression with GEE
Multiple categories	*Patient-provider communication* (The primary units of turns)	Multinomial logistic regression with GEE

Finally, Aim 4 (to quantify the impact of patient-provider communication/clinical discussion content with patient-centered outcomes) examines Paths E and F in our conceptual model. The analyses will be carried out in steps similar to those in Aim 3. In Step 1, we will identify covariates associated with patient-centered outcomes by computing correlations among all aspects of patient-provider communication/clinical discussion topics and factors assessed in the patient baseline survey, the provider baseline survey, and the provider post-encounter survey. In Step 2, we will compute partial correlations between all aspects of patient-provider communication/clinical discussion topics identified in Aims 1 and 2 and patient-centered outcomes while controlling for covariates identified in Step 1. In Step 3, we will conduct a linear regression with GEE and Bonferroni correction for each self-reported outcome (e.g., trust, satisfaction, therapeutic alliance) and a logistic regression with GEE for behavioral outcome (genetic testing completion). All regressions will include the same set of predictors (aspects of patient-provider communication/clinical discussion topics that remain significant in Step 2, and care modality) and covariates. Aspects of patient-provider communication/clinical discussion topics that remain significant in Step 3 are considered as potentially important intervention points for reducing racial disparities in patient-centered outcomes.

## Discussion

We acknowledge that the role of counselor bias in racial healthcare disparities may be statistically small [27, 30]. However, even statistically small effects can have significant societal impact when a large number of people are affected, [95, 96] as in the case of Black patients interacting with White genetic counselors. We also acknowledge that counselor bias is one of many multilevel determinants of racial disparities. Additionally, individual bias is closely tied to systemic racism [97, 98]. Thus, addressing counselor bias—an individual level factor—is not sufficient to eliminate racial disparities in the genetic counseling process. Yet, structural-level factors, including systemic racism and the effort to diversify genetic counselors, are highly resistant and slow to change, requiring significant time and financial resources. Given the urgency of the current state of racial cancer disparities, it is critical to address modifiable factors that can produce desired outcomes immediately, including counselors’ bias-associated behaviors, while continuing to address structural-level factors.

We emphasize bias-related behaviors, not bias per se, because changing fundamental attitudes and beliefs that are deeply ingrained in our society and have perpetuated systemic racism for centuries [97, 98] is a challenge, requiring considerable investment of time and resources [57, 99–101]. Time and volume constraints in the US healthcare system make it difficult for genetic counselors to use evidence-based strategies to reduce bias (stereotype replacement, counter-stereotypical imaging, individuation) [99, 102]. Our conceptual model highlights patient-provider communication and clinical discussions as the mediators linking counselor bias to patient-centered outcomes, suggesting that a viable alternative approach is to train genetic counselors to better *manage their bias-associated behaviors* during encounters with Black patients [22, 103, 104]. In fact, counselor communication behaviors and clinical discussions are an important target point in future interventions because they are both being taught in genetic counseling programs [105]. Thus, interventions that are designed to help counselors manage their bias-associated behaviors can be delivered using the existing infrastructure, which is both cost-effective and efficient.

Once we identify specific counselor communication behaviors associated with patient-centered outcomes, we can design interventions to help genetic counselors adapt their communication behaviors accordingly. Likewise, once we identify specific clinical discussion topics directly associated with patient-centered outcomes, interventions can be designed to provide genetic counselors with a list of topics that need to be discussed and a list of topics that may be omitted during pre-testing cancer genetic counseling encounters. We are not arguing that changing communication behaviors or clinical discussions is easy; indeed, changing those particular behaviors requires effort and practice. However, communication behaviors and clinical discussions are relatively modifiable, and that is why they are being taught in genetic counseling programs [105]. Indeed, genetic counselors and training programs are heavily invested in improving training in this area. The Accreditation Council for Genetic Counseling (2019) standards state that MS Genetic Counseling curricula must include content related to “multicultural sensitivity and competence.” [106] A recent study found that, 90% of genetic counseling graduate programs provide opportunities for diversity, inclusion, cultural competency, and equity (DICE) training, and 68% require it [11]. Our research will lay the foundation for interventional research to manage counselor bias-associated behaviors and further inform DICE training.

## Data Availability

Not applicable for this protocol paper. Data from the project will be available mainly through presentations at scientific conferences, publications, and speaking engagements.

## Abbreviations

MS: Master of Science
HIPAA: Health Insurance Portability and Accountability Act
QR code: Quick response code
RIAS: Rotor Interaction Analysis System
LIWC: Linguistic Inquiry and Word Count
ICC: Intraclass correlation coefficient
NCI: National Cancer Institute
IAT: Implicit Association Test
BMI: Body mass index
GOS: Genomics Outcomes Scale
ATGT: Attitudes Toward Genetic Testing
DCS: Decisional Conflict Scale
WAI-SR: Working Alliance Inventory-Short Form Revised
GCSS: Genetic Counseling Satisfaction Scale
GEE: Generalized estimating equation
DICE: Diversity, inclusion, cultural competency, and equity

## References

[1] American Cancer Society (2019). Cancer facts & figures for African Americans 2019–2021.

[2] Alvarado M, Tiller GE, Chung J, Haque R. Prevalence of mutations in a diverse cohort of 3162 women tested via the same multigene cancer panel in a managed care health plan. J Community Genet. 2020. 10.1007/s12687-020-00456-6 PMC7295891 32096056

[3] Jones T, Trivedi MS, Jiang X, Silverman T, Underhill M, Chung WK et al. Racial and ethnic differences in BRCA1/2 and Multigene Panel Testing among young breast Cancer patients. J Cancer Educ. 2019. 10.1007/s13187-019-01646-8 PMC7293107 31802423

[4] World Health Organization. Cancer control: knowledge into action : WHO guide for effective programmes. In: Promotion DoCDaH, editor. Geneva, Switzerland2007.

[5] Task F, Resta R, Biesecker BB, Bennett RL, Blum S, Hahn SE, National Society of Genetic Counselors’ Definition (2006). A new definition of genetic counseling: National Society of genetic counselors’ Task Force report. J Genet Couns.

[6] Cragun D, Weidner A, Lewis C, Bonner D, Kim J, Vadaparampil ST (2017). Racial disparities in BRCA testing and cancer risk management across a population-based sample of young breast cancer survivors. Cancer.

[7] Muller C, Lee SM, Barge W, Siddique SM, Berera S, Wideroff G (2018). Low referral rate for genetic testing in racially and ethnically diverse patients despite Universal Colorectal Cancer Screening. Clin Gastroenterol Hepatol.

[8] Peterson JM, Pepin A, Thomas R, Biagi T, Stark E, Sparks AD et al. Racial disparities in breast cancer hereditary risk assessment referrals. J Genet Couns. 2020. 10.1002/jgc4.1250 32196827

[9] Southwick SV, Esch R, Gasser R, Cragun D, Redlinger-Grosse K, Marsalis S (2020). Racial and ethnic differences in genetic counseling experiences and outcomes in the United States: a systematic review. J Genet Couns.

[10] Nikolaidis C, Duquette D, Mendelsohn-Victor KE, Anderson B, Copeland G, Milliron KJ (2019). Disparities in genetic services utilization in a random sample of young breast cancer survivors. Genet Med.

[11] Channaoui N, Khan A, Wiesman C, Bui K, Cunningham M, Brown K (2020). Summary report of the 2019 diversity and inclusion Task Force of the National Society of genetic counselors. J Genet Couns.

[12] Colleges AoAM. Figure 18. Percentage of all active physicians by race/ethnicity, 20182020 September 19., 2020. Available from: https://www.aamc.org/data-reports/workforce/interactive-data/figure-18-percentage-all-active-physicians-race/ethnicity-2018.

[13] Cooper LA, Roter DL, Johnson RL, Ford DE, Steinwachs DM, Powe NR (2003). Patient-centered communication, ratings of Care, and concordance of patient and physician race. Ann Intern Med.

[14] Cooper-Patrick L, Gallo JJ, Gonzales JJ, Vu HT, Powe NR, Nelson C (1999). Race, gender, and Partnership in the patient-physician relationship. JAMA.

[15] Johnson RL, Roter D, Powe NR, Cooper LA (2004). Patient Race/Ethnicity and quality of patient–physician communication during medical visits. Am J Public Health.

[16] Oliver MN, Goodwin MA, Gotler RS, Gregory PM, Stange KC (2001). Time use in clinical encounters: are african-american patients treated differently?. J Natl Med Assoc.

[17] Siminoff LA, Graham GC, Gordon NH (2006). Cancer communication patterns and the influence of patient characteristics: disparities in information-giving and affective behaviors. Patient Educ Couns.

[18] Wilson TD, Lindsey S, Schooler TY (2000). A model of dual attitudes. Psychol Rev.

[19] Dovidio JF, Gaertner SL (2010). Intergroup bias.

[20] Dovidio JF, Kawakami K, Gaertner L (2002). Implicit and explicit prejudice and interacial interaction. J Personal Soc Psychol.

[21] Hoffman KM, Trawalter S (2016). Assumptions about life hardship and pain perception. Group Processes & Intergroup Relations.

[22] Hagiwara N, Duffy C, Quillin J (2023). Implicit and explicit racial prejudice and stereotyping toward Black (vs. White) Americans: the prevalence and variation among genetic counselors in North America. J Genet Couns.

[23] Hagiwara N, Dovidio JF, Stone J, Penner LA (2020). Applied Racial/Ethnic Healthcare Disparities Research using implicit measures. Soc Cogn.

[24] Cooper LA, Roter DL, Carson KA, Beach MC, Sabin JA, Greenwald AG (2012). The associations of clinicians’ implicit attitudes about race with medical visit communication and patient ratings of interpersonal care. Am J Public Health.

[25] Hagiwara N, Dovidio JF, Eggly S, Penner LA (2016). The effects of racial attitudes on affect and engagement in racially discordant medical interactions between non-black physicians and black patients. Group Processes & Intergroup Relations: GPIR.

[26] Hagiwara N, Penner LA, Gonzalez R, Eggly S, Dovidio JF, Gaertner SL (2013). Racial attitudes, physician-patient talk time ratio, and adherence in racially discordant medical interactions. Soc Sci Med.

[27] Hagiwara N, Slatcher RB, Eggly S, Penner LA (2017). Physician racial Bias and Word Use during racially discordant medical interactions. Health Commun.

[28] Penner LA, Dovidio JF, Gonzalez R, Albrecht TL, Chapman R, Foster T et al. The effects of oncologist implicit racial bias in racially discordant oncology interactions. J Clin Oncol. 2016. 10.1200/JCO.2015.66.3658 PMC5012663 27325865

[29] Stewart MA, Brown JB, Donner A, McWhinney I, Oates J, Weston W (2000). The impact of patient-centered care on outcomes. J Fam Pract.

[30] Street RL, Makoul G, Arora NK, Epstein RM (2009). How does communication heal? Pathways linking clinician-patient communication to health outcomes. Patient Educ Couns.

[31] Penner LA, Dovidio JF, West TW, Gaertner SL, Albrecht TL, Dailey RK (2010). Aversive racism and medical interactions with black patients: a field study. J Exp Soc Psychol.

[32] Calabrese SK, Earnshaw VA, Magnus M, Hansen NB, Krakower DS, Underhill K (2018). Sexual stereotypes ascribed to Black Men who have sex with men: an intersectional analysis. Arch Sex Behav.

[33] Hoffman KM, Trawalter S, Axt JR, Oliver MN (2016). Racial bias in pain assessment and treatment recommendations, and false beliefs about biological differences between blacks and whites. Proc Natl Acad Sci USA.

[34] van Ryn M, Burke J (2000). The effect of patient race and socio-economic status on physicians’ perceptions of patients. Soc Sci Med.

[35] Lowe C, Beach MC, Roter DL (2020). Individuation and implicit racial bias in genetic counseling communication. Patient Educ Couns.

[36] Lowe CL, Beach MC, Roter DL (2020). Genetic counselor implicit bias and its effects on cognitive and affective exchanges in racially discordant simulations. J Genet Couns.

[37] Fiscella K, Franks P, Srinivasan M, Kravitz RL, Epstein R (2007). Ratings of physician communication by real and standardized patients. Ann Fam Med.

[38] Athens BA, Caldwell SL, Umstead KL, Connors PD, Brenna E, Biesecker BB (2017). A systematic review of Randomized controlled trials to assess outcomes of genetic counseling. J Genet Couns.

[39] Cragun D, Zierhut H (2018). Development of FOCUS-GC: Framework for Outcomes of Clinical Communication Services in genetic counseling. J Genet Couns.

[40] Senter LA, Austin J, Choe M, Harris S, Linnenbringer E, MacFarlane I et al. Advancing the Genetic Counseling Profession through Research: Identification of Priorities by the NSGC Research Task Force. J Genet Couns. In Press. 10.1002/jgc4.1330 PMC8279295 32969094

[41] Chen YC, Li IC (2009). Effectiveness of interventions using empowerment concept for patients with chronic disease: a systematic review. JBI Libr Syst Rev.

[42] Mohottige D, Boulware LE (2020). Trust in American Medicine: a call to action for Health Care Professionals. Hastings Cent Rep.

[43] Bradbury AR, Patrick-Miller L, Long J, Powers J, Stopfer J, Forman A (2015). Development of a tiered and binned genetic counseling model for informed consent in the era of multiplex testing for cancer susceptibility. Genet Med.

[44] Scheinberg T, Young A, Woo H, Goodwin A, Mahon KL, Horvath LG. Mainstream consent programs for genetic counseling in cancer patients: a systematic review. Asia-Pac J Clin Oncol. 2020. 10.1111/ajco.13334 32309911

[45] GoPro. [Available from: https://gopro.com/en/us/shop/mounts-accessories/the-remote/ARMTE-003.html?clickId=4478678330&utm_campaign=159404&utm_content=8-12501&utm_medium=affiliate&utm_source=pepperjam.

[46] TechSmith®. Camtasia® [Available from: https://www.techsmith.com/video-editor.html.

[47] Roter D, Larson S (2002). The Roter interaction analysis system (RIAS): utility and flexibility for analysis of medical interactions. Patient Educ Couns.

[48] Schaa KL, Roter DL, Biesecker BB, Cooper LA, Erby LH (2015). Genetic counselors’ implicit racial attitudes and their relationship to communication. Health Psychology: Official Journal of the Division of Health Psychology American Psychological Association.

[49] Ambady N, Rosenthal R (1992). Thin slices of expressive behavior as predictors of interpersonal consequences: a meta-analysis. Psychol Bull.

[50] Ambady N, Bernieri FJ, Richeson JA. Toward a histology of social behavior: Judgmental accuracy from thin slices of the behavioral stream. Advances in Experimental Social Psychology. 32: Academic Press; 2000. p. 201 – 71.

[51] Weisbuch M, Ambady N. 228Thin-Slice vision. In: Adams RB, Ambady N, Nakayama K, Shimojo S, editors. The Science of Social Vision. Oxford University Press; 2010. p. 0.

[52] Pratto F, Sidanius J, Stallworth LM, Malle BF (1994). Social dominance orientation: a personality variable predicting social and political attitudes. J Personal Soc Psychol.

[53] Sidanius J, Pratto F. Social dominance: An intergroup theory of social hierarchy and oppression. New York, NY, US: Cambridge University Press; 1999. x, 403-x, p.

[54] Berry Mendes W, Gray HM, Mendoza-Denton R, Major B, Epel ES (2007). Why Egalitarianism might be good for your health: physiological thriving during Stressful Intergroup encounters. Psychol Sci.

[55] Page-Gould E, Mendoza-Denton R, Tropp LR (2008). With a little help from my cross-group friend: reducing anxiety in intergroup contexts through cross-group friendship. J Personal Soc Psychol.

[56] Stephan WG, Stephan CW (1985). Intergroup anxiety. J Soc Issues.

[57] Gaertner SL, Dovidio JF (2000). Reducing intergroup bias: the common ingroup identity model.

[58] Plant EA, Devine PG (2003). The antecedents and implications of interracial anxiety. Pers Soc Psychol Bull.

[59] Schlenker BR, Leary MR (1982). Social anxiety and self-presentation: a conceptualization model. Psychol Bull.

[60] Dunbar NE, Burgoon JK (2005). Measuring nonverbal dominance. The sourcebook of nonverbal measures: going beyond words.

[61] Alafifi M. Presidential Manifestation of Verbal Dominance: A discourse analysis of conversational dominance strategies employed by Joe Biden and Donald Trump [Student thesis]2021.

[62] Baker MA (1991). Gender and Verbal Communication in Professional settings: a review of Research. Manage Communication Q.

[63] Dunbar NE, Burgoon JK (2005). Perceptions of power and interactional dominance in interpersonal relationships. J Social Personal Relationships.

[64] Glauser MJ (1984). Self-esteem and communication tendencies: an analysis of four Self-Esteem/Verbal dominance personality types. Psychol Record.

[65] Park J, Beach MC, Han D, Moore RD, Korthuis PT, Saha S (2020). Racial disparities in clinician responses to patient emotions. Patient Educ Couns.

[66] Tausczik YR, Pennebaker JW (2010). The psychological meaning of words: LIWC and computerized text analysis methods. J Lang Social Psychol.

[67] Pennebaker JW, Boyd RL, Jordan KN, Blackburn KG, editors. The Development and Psychometric Properties of LIWC20152015.

[68] Syed M, Nelson SC. Guidelines for Establishing Reliability When Coding Narrative Data. Emerging adulthood (Thousand Oaks, CA). 2015;3(6):375 – 87.

[69] O’Connor C, Joffe H (2020). Intercoder reliability in qualitative research: debates and practical guidelines. Int J Qualitative Methods.

[70] PCGE Board. PDQ Cancer Genetics Risk Assessment and Counseling Bethesda, MD: National Cancer Institute. ; 2020 [updated 05/08/2020. Available from: https://www.cancer.gov/about-cancer/causes-prevention/genetics/risk-assessment-pdq.

[71] Stemler SE. A Comparison of Consensus, Consistency, and Measurement Approaches to Estimating Interrater Reliability. Practical Assessment, Research, and Evaluation. 2004;9(Article 4).

[72] Greenwald AG, McGhee DE, Schwartz JLK (1998). Measuring individual differences in implicit cognition: the implicit association test. J Personal Soc Psychol.

[73] Greenwald AG, Poehlman TA, Uhlmann EL, Banaji MR (2009). Understanding and using the implicit association test: III. Meta-analysis of predictive validity. J Personal Soc Psychol.

[74] Greenwald AG, Nosek BA, Banaji MR (2003). Understanding and using the implicit association test: I. An improved scoring algorithm. J Personal Soc Psychol.

[75] Campbell DT (1971). White attitudes toward black people.

[76] Axt JR (2017). The best way to measure explicit racial attitudes is to ask about them. Social Psychol Personality Sci.

[77] Green AR, Carney DR, Pallin DJ, Ngo LH, Raymond KL, Iezzoni LI (2007). Implicit bias among physicians and its predictions of thrombolysis decisions for Black and White patients. J Gen Intern Med.

[78] Oliver MN, Wells KM, Joy-Gaba JA, Hawkins CB, Nosek BA (2014). Do physicians’ implicit views of African Americans affect clinical decision making?. J Am Board Family Medicine: JABFM.

[79] Brondolo E, Kelly KP, Coakley V, Gordon T, Thompson S, Levy E (2005). The Perceived ethnic discrimination questionnaire: development and preliminary validation of a Community Version. J Appl Soc Psychol.

[80] Hagiwara N, Alderson CJ, McCauley JM (2015). We get what we deserve: the belief in a just world and its health consequences for blacks. J Behav Med.

[81] Hagiwara N, Alderson CJ, Mezuk B (2016). Differential Effects of Personal-Level vs Group-Level racial discrimination on Health among Black Americans. Ethn Dis.

[82] Hall MA, Camacho F, Dugan E, Balkrishnan R (2002). Trust in the medical profession: conceptual and measurement issues. Health Serv Res.

[83] Hall MA, Zheng B, Dugan E, Camacho F, Kidd KE, Mishra A (2002). Measuring patients’ trust in their primary care providers. Med care Res Review: MCRR.

[84] Ware JE, Snyder MK, Wright W (1976). Development and validation of scales to measure patient satisfaction with medical care services.

[85] Grant PE, Pampaka M, Payne K, Clarke A, McAllister M (2019). Developing a short-form of the genetic counselling outcome scale: the Genomics Outcome Scale. Eur J Med Genet.

[86] McAllister M, Wood AM, Dunn G, Shiloh S, Todd C (2011). The genetic counseling outcome scale: a new patient-reported outcome measure for clinical genetics services. Clin Genet.

[87] Bouhnik AD, N’Diaye K, Evans DG, Harris H, Tibben A, van Asperen C (2017). Validation of a scale for assessing attitudes towards outcomes of genetic cancer testing among primary care providers and breast specialists. PLoS ONE.

[88] O’Connor AM (1995). Validation of a Decisional Conflict Scale. Med Decis Making.

[89] Paap D, Dijkstra PU (2017). Working Alliance Inventory-Short Form revised. J Physiother.

[90] DeMarco TA, Peshkin BN, Mars BD, Tercyak KP (2004). Patient satisfaction with cancer genetic counseling: a psychometric analysis of the genetic counseling satisfaction scale. J Genet Couns.

[91] Conley CC, Garcia JD, Radford C, Reich RR, Monteiro AN, Pal T et al. Acceptability and outcomes of multigene panel testing among young black breast cancer survivors. Breast J. 2020. 10.1111/tbj.13848 PMC7578013 32319712

[92] Tutty E, Petelin L, McKinley J, Young M-A, Meiser B, Rasmussen VM (2019). Evaluation of telephone genetic counselling to facilitate germline BRCA1/2 testing in women with high-grade serous ovarian cancer. Eur J Hum Genet.

[93] Dunn OJ. Estimation of the Medians for Dependent Variables. 1959:192-7.

[94] Dunn OJ (1961). Multiple comparisons among means. J Am Stat Assoc.

[95] Greenwald AG, Banaji MR, Nosek BA (2015). Statistically small effects of the Implicit Association Test can have societally large effects. J Personal Soc Psychol.

[96] Rosenthal R (1990). How are we doing in soft psychology?. Am Psychol.

[97] Payne BK, Vuletich HA, Brown-Iannuzzi JL. Historical roots of implicit bias in slavery. Proceedings of the National Academy of Sciences. 2019;116(24):11693. 10.1073/pnas.1818816116 PMC6575576 31138682

[98] Payne BK, Vuletich HA, Lundberg KB (2017). The Bias of crowds: how implicit Bias Bridges Personal and systemic prejudice. Psychol Inq.

[99] Devine PG, Forscher PS, Austin AJ, Cox WTL (2012). Long-term reduction in implicit race bias: a prejudice habit-breaking intervention. J Exp Soc Psychol.

[100] Rydell RJ, McConnell AR (2006). Understanding implicit and explicit attitude change: a systems of reasoning analysis. J Personal Soc Psychol.

[101] Wood W (2000). Attitude change: persuasion and social influence. Ann Rev Psychol.

[102] Stone J, Moskowitz GB, Zestcott CA, Wolsiefer KJ (2020). Testing active learning workshops for reducing implicit stereotyping of Hispanics by majority and minority group medical students. Stigma and Health.

[103] Hagiwara N, Elston Lafata J, Mezuk B, Vrana SR, Fetters MD (2019). Detecting implicit racial bias in provider communication behaviors to reduce disparities in healthcare: Challenges, solutions, and future directions for provider communication training. Patient Educ Couns.

[104] Hagiwara N, Kron FW, Scerbo MW, Watson GS (2020). A call for grounding implicit bias training in clinical and translational frameworks. Lancet (London England).

[105] Accreditation Council for Genetic Counseling. Practice-Based Competencies for Genetic Counselors. 2019. 10.1007/s10897-016-9984-3 27333894

[106] Accreditation Council for Genetic Counseling. Standards of Accreditation for Graduate Programs in Genetic Counseling2019 8/21/2023. Available from: https://www.gceducation.org/wp-content/uploads/2022/04/https___www.gceducation.org_wp-content_uploads_2020_06_Revised-Standards-of-Accreditation_Final_Effective-10.1.19__6.17.20-Comp-Date-Rev-5.1-to-6.15.pdf.

